# Results and reflections from the PROfiling Consortium on Antibody Repertoire and Effector functions in kidney transplantation: A mini‐review

**DOI:** 10.1111/tan.13581

**Published:** 2019-06-18

**Authors:** Elena G. Kamburova, Andries Hoitsma, Frans H. Claas, Henny G. Otten, On behalf of the PROCARE Consortium

**Affiliations:** ^1^ Laboratory of Translational Immunology, University Medical Center Utrecht Utrecht University Utrecht The Netherlands; ^2^ Dutch Organ Transplant Registry (NOTR) Dutch Transplant Foundation (NTS) Leiden The Netherlands; ^3^ Department of Immunohematology and Blood Transfusion Leiden University Medical Center Leiden The Netherlands

**Keywords:** HLA antibodies, HLA epitope, kidney transplantation, non‐HLA antibodies

## Abstract

Kidney transplantation is the best treatment option for patients with end‐stage renal disease (ESRD). The waiting time for a deceased donor kidney in the Netherlands is approximately 3 years. Mortality among patients on the waiting list is high. The aim of the PROCARE consortium (PROfiling Consortium on Antibody Repertoire and Effector functions) was to decrease the waiting time by providing a matching algorithm yielding a prolonged graft survival and less HLA‐immunization compared with the currently used Eurotransplant Kidney allocation system. In this study, 6097 kidney transplants carried out between January 1995 and December 2005 were re‐examined with modern laboratory techniques and insights that were not available during that time period. In this way, we could identify potential new parameters that can be used to improve the matching algorithm and prolong graft survival. All eight University Medical Centers in the Netherlands participated in this multicenter study. To improve the matching algorithm, we used as central hypothesis that the combined presence of class‐I and ‐II single‐antigen bead (SAB)‐defined donor‐specific HLA antibodies (DSA) prior to transplantation, non‐HLA antibodies, the number of B‐ and/or T‐cell epitopes recognized on donor HLA, and specific polymorphisms in effector mechanisms of IgG were associated with an increased risk for graft failure. The purpose of this article is to relate the results obtained from the PROCARE consortium study to other studies published in recent years. The clinical relevance of SAB‐defined DSA, complement‐fixing DSA, non‐HLA antibodies, and the effector functions of (non)‐HLA‐antibodies will be discussed.

## 1 INTRODUCTION

Kidney transplantation is the best treatment option for patients with end‐stage renal disease (ESRD). Currently, approximately 650 Dutch patients are registered on the active waiting list of Eurotransplant. The mean waiting time for a deceased donor kidney in the Netherlands is approximately 2.5 years. Patients with severe kidney failure are fully dependent on dialysis, which limits their quality of life. In 2017, 82 ESRD patients died because a donor kidney was not available in time.^1^ In 2014, all eight University Medical Centers in the Netherlands have joint forces in the PROfiling Consortium on Antibody Repertoire and Effector (PROCARE) consortium to redefine the matching strategy currently used for organ allocation by performing a comprehensive analysis of various immunological risk factors for rejection and graft loss.

The aim of the PROCARE study was to improve the Dutch matching algorithm, and the central hypothesis of this study was that the combined presence of class‐I and ‐II single‐antigen bead‐defined donor‐specific HLA antibodies (DSA) present prior to transplantation, non‐HLA antibodies, the number of B‐ and/or T‐cell epitopes recognized on donor HLA, and specific polymorphisms in effector mechanisms of IgG were associated with an increased risk for graft failure. Weighed inclusion of these results could be used to improve the matching algorithm.

### 1.1 Collection of clinical data

**Figure 1:**
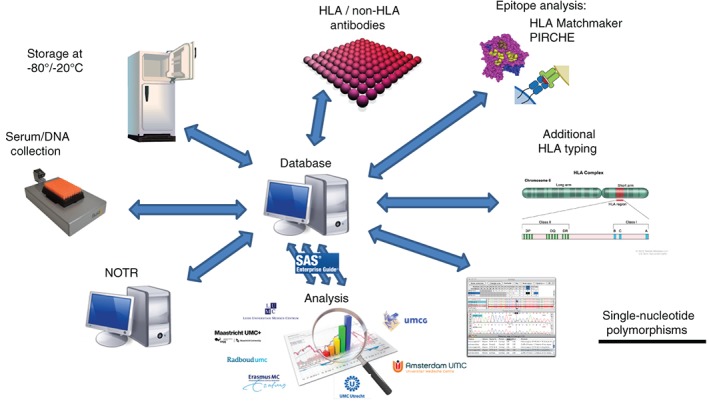
Overview of the PROfiling Consortium on Antibody Repertoire and Effector (PROCARE) ICT infrastructure with the different clinical and laboratory data. NOTR, Dutch Organ Transplant Registry; PIRCHE, Predicted Indirectly ReCognizable HLA Epitopes

**Figure 2:**
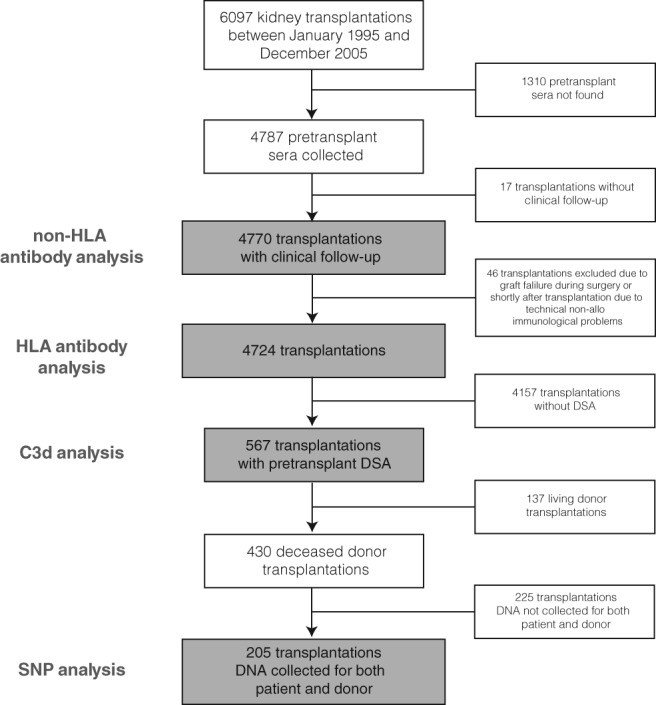
Flow chart for the inclusion and exclusion of transplantations for the different analyses. DSA, donor‐specific HLA antibodies; SNP, single nucleotide polymorphisms

Evidence‐based recommendations aimed to improve the kidney transplantation allocation system, must be based on large amounts of solid, shared, and reproducible data as has been shown in multiple large‐case studies..^2^, ^3^, ^4^, ^5^, ^6^, ^7^ All data from the PROCARE consortium are located in a central database which is accessible for all participants enabling reproduction of published data (Figure 1). Clinical and laboratory data of 6097 kidney transplants performed between January 1995 and December 2006 from all eight transplant centers in the Netherlands were included. At the start of the project, all clinical variables required for the study (listed in Box 1) were extracted from the Dutch Organ Transplant Registry (NOTR) and included in above‐mentioned infrastructure. However, the NOTR was established in 2002, so only data was included since that period. The completeness of data, obtained after 2002 for major items such as graft failure, patient death was almost 100%. For other information, such as creatinine, number of rejections, proteinuria, etc. the completeness was about 80%. Some centers also had details registered of transplants performed before 2002. The completeness for a large number of other items of that period was about 40%. The examined period of the requested study consists also for an important part of the period before 2002 and for a reliable study, data needed to be supplemented. From the start of the PROCARE study, all centers were provided with information on missing data. Each center re‐examined the transplant cases involved and supplemented missing data to the consortium database within 1 year after the start of the study. The data mentioned in Box 1 was completed by the eight centers up to 98%. From 1995 to 2005, a total number of 6097 kidney transplantation were performed from which 4770 could be included in a non‐HLA antibody study consisting of 1496 living and 3274 deceased donor transplantations. In the study on a study on the effect of DSA on long‐term graft survival, 4724 patients were included with 3237 deceased‐ and 1487 living‐donor kidney transplantations. Of these transplantations, 567 were found to have pre‐transplant DSA (with 130 living and 430 deceased donors) which were included in a study on the relevance for C3D fixing luminex defined DSA (Figure 2).

**BOX 1:** Clinical endpoints and covariates

Primary endpoint	Secondary endpoints
Graft failure	The number of rejections
	Time to first rejection
	Time to first biopsy
	Creatinine levels^a^
	Proteinuria^a^
	Incidence of delayed graft function
Recipient covariates	Donor covariates
Age at transplant	Age at death
Sex	Sex
No. of transplants	Type of donor (living, [non‐]heart beating)
Date and cause of death	Cause of death
% HLA immunization	HLA mismatches
The type (or change) of Immunosuppression^a^	Cold ischemia time
Use of induction therapy	

a Follow‐up at 3 months, 12 months and yearly thereafter for at least 10 years.

In registries, such as the UNOS (https://unos.org/), CTS (http://www.ctstransplant.org/), UKRR (https://www.renalreg.org/), comparable databases are maintained containing basic demographic data supplemented with high‐quality laboratory and/or clinical data. Information from these databases can be requested for research purposes, such as validation studies. This has led to multiple publications with translational objectives, but especially clinically and laboratory data included from the early days of kidney transplantation were not as complete compared with the recent decade. This was also the case for the PROCARE study.

The PROCARE study allowed analysis of outcome of transplantations, with donor acceptance unbiased by results and knowledge that were post‐hoc obtained with techniques used in PROCARE study. Recommendations reflecting the impact of parameters from that study are currently well appreciated.^8^, ^9^, ^10^, ^11^, ^12^

## 2 CLINICAL RELEVANCE OF ANTI‐HLA ANTIBODIES DETECTED BY SINGLE ANTIGEN BEAD ASSAYS IN KIDNEY TRANSPLANTATION

### 2.1 Anti‐HLA antibodies

Pre‐transplant DSA are associated with impaired kidney graft survival, while the clinical relevance of non‐donor specific anti‐HLA antibodies (nDSA) is more controversial. Some studies have shown that nDSA have a negative impact on graft survival and antibody‐mediated rejection,^13^, ^14^ while others did not find an inverse association between pre‐transplant nDSA and graft survival.^15^, ^16^, ^17^, ^18^ To eliminate donor and era‐dependent factors, we performed a post‐hoc paired kidney graft analysis using the PROCARE cohort. Among 3237 deceased donor transplantations, we identified 192 pairs with one recipient nDSA positive (against class I and/or II) and the other without anti‐HLA antibodies. For the patients with nDSA against either class I or II, graft survival did not significantly differ compared to patients without anti‐HLA antibodies.^19^ Only in patients with both nDSA classes I and II, there was a trend towards a lower graft survival. This paired kidney analysis confirmed that the presence of pre‐transplant DSA in deceased donor transplantations is a risk factor for graft loss, whereas nDSA in general are not associated with a lower graft survival.

### 2.2 Donor‐specific anti‐HLA antibodies

**Figure 3:**
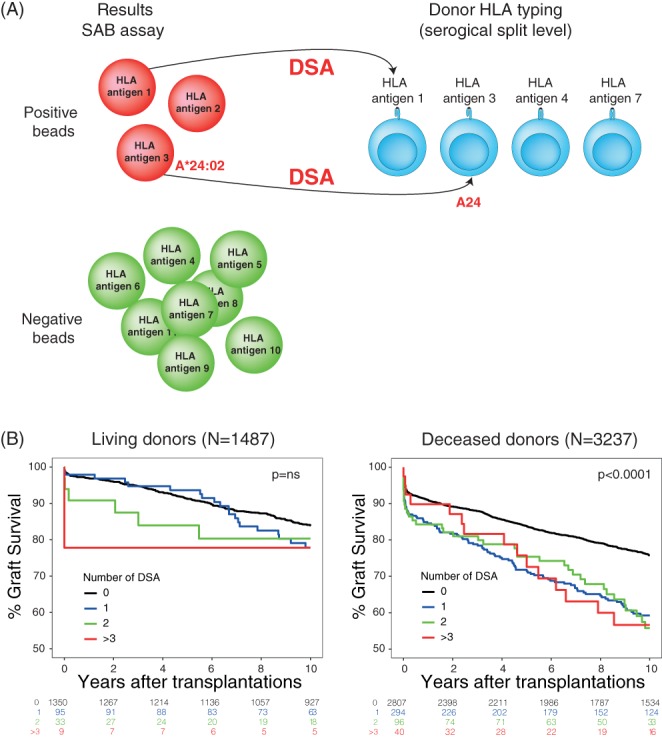
A, Assignment of donor‐specific antibodies (DSA) using the single‐antigen bead (SAB) assay and serological split‐level donor HLA‐A, ‐B, ‐DRB1, −DQ typing. If a bead is positive (ie, A*24:02) and this antigen was also present on the donor HLA (ie, A24), we assigned this as a DSA. (B) Death‐censored 10‐year graft survival stratified according to the number of pre‐transplant DSA for 1487 living‐donor transplantations and 3237 deceased‐donor transplantations

The relevance of these pre‐transplant donor‐antibodies was established in deceased donor kidney transplantation. Luminex‐defined DSA, detectable in pre‐transplant sera with a negative CDC cross‐match, was one important identified parameter indicating an increased immunological risk for rejection and allograft loss.^20^, ^21^ In most studies, DSA were defined as antibodies to donor HLA defined at the serological split level whereas others used a higher resolution of DSA assignment enabling the detection of the number of HLA epitopes recognized.^22^ Results from large‐scale cohorts were not reported in patients without desensitization treatment transplanted with kidneys from living donors. A study on long‐term graft survival in 3237 deceased and 1487 living donor kidney transplants showed that SAB‐defined DSA appear to be very relevant in transplantation deceased but not with living donor kidneys.^11^ Especially, the combination of DSA against classes I and II appeared to be related to a poor prognosis, although the numbers present in the living cohort were not sufficient to assess this. This study showed that with this new information, prior to transplantation the chance of success of a kidney transplant can be estimated much better than before, especially since these DSA were not demonstrable by classic complement‐dependent cross‐match technique. Interestingly, the number of DSA apparently was relevant for risk stratification of graft loss only after deceased donor but not after living donor kidney transplantation described in Reference 11 as text (Figure 3).

### 2.3 B‐cell recognition of donor HLA epitopes

After the introduction of molecular typing techniques, including sequence‐based typing, the molecular nature of the targets of HLA alloantibodies became clearer. In the meantime, more than 13 000 different HLA class I alleles have been described (http://hla.alleles.org/nomenclature/stats.html) whereas the polymorphism of these HLA alleles with respect to antibody reactivity is based on approximately 180 crucial amino acid variations (epitopes/eplets, https://www.epvix.com.br/). HLA molecules can be considered as patchwork molecules consisting of a number of epitopes.^23^ Furthermore, epitopes are often shared between different HLA molecules, which is the reason why the immunogenicity of an HLA mismatch is dependent on the own HLA antigens of the recipient. A particular HLA mismatch may have many potential antibody epitopes in one patient but no foreign epitopes in another patient. The HLAMatchmaker algorithm developed by Rene Duquesnoy in Pittsburgh in close collaboration with the group in Leiden has been instrumental for the identification of the most important antibody epitopes.^24^ Also, other HLA molecular methods to define mismatches such as analysis for amino acid mismatch and electrostatic mismatch between donor and recipient HLA have been developed and validated.^25^ Each method is an improvement over the classic HLA mismatch calculation (0, 1 or 2 mismatches per locus) and improves improved risk stratification with regard to development of de‐novo DSA.^25^

**Figure 4:**
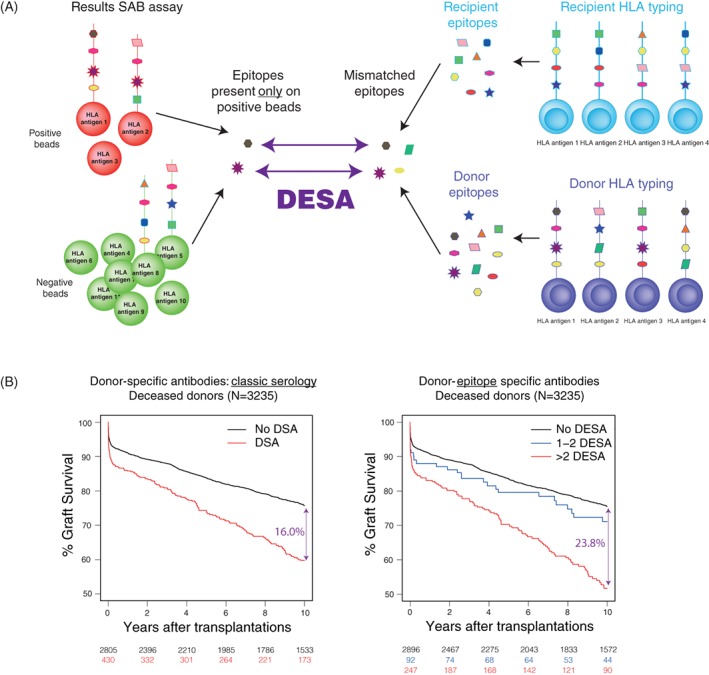
Assignment of donor‐epitope specific antibodies (DESA) using the single‐antigen bead (SAB) assay and the most likely high‐resolution HLA‐A, ‐B, ‐C, ‐DRB1, ‐DRB3‐5, −DQA1, ‐DQB1 typing for donor and recipient. First, the HLA typing's were converted into epitopes. Next, the recipient and donor epitopes were compared, and epitopes of the donor HLA that were not present on the recipient HLA were assigned as mismatched. Also, the results of the SAB assay were converted into epitopes. If an epitope, that was only present on positive beads, was one of the mismatched epitopes, we assigned this as a DESA

In order to assess the clinical relevance of DSA assessment according to classical serology vs epitope/eplet based definition, donor‐epitope specific HLA antibodies (DSA), we upscaled serological typing results from patients and donors to high‐resolution data using HLA haplotype frequency tables, and converted Luminex‐defined HLA‐antibody patterns into recognized epitopes on donor HLA.^26^, ^27^ When DSA was defined by classic serology, a graft survival difference of 16% was observed between patients with and without pre‐transplant DSA transplanted with kidneys derived from deceased donors^11^ (Figure 4A). When DSA was defined according to epitopes recognized on donor HLA, this difference in graft survival increased to almost 24%, indicating the added value of epitope‐based definition of DSA (Figure 4B, unpublished data). These data are in agreement with other studies comparing serological vs eplet‐based DSA assignment.^28^, ^29^

### 2.4 T‐cell recognition of donor HLA class‐II epitopes

Cognate T‐cell help is required for proliferation and differentiation of antigen‐specific naïve B‐cells into IgG‐producing cells. During this process, mismatched HLA is internalized by B‐cells, processed, and HLA‐derived epitopes can subsequently be loaded onto HLA class II molecules on the surface of B‐cells. In case of production of IgG anti‐HLA antibodies, mismatched HLA epitopes have to be recognized by recipient T‐cells. T‐cells may also directly harm the transplanted kidney after recognition. In this process, indirect recognition of HLA may also play a role. The predicted indirectly ReCognizable HLA epitopes presented by recipient HLA class II (PIRCHE‐II) algorithm is able to predict HLA‐mismatch derived T‐cell epitopes by quantifying the number of mismatched donor HLA‐derived peptides that can be presented on HLA class II molecules of the recipient, designated as PIRCHE‐II. The number of PIRCHE‐II has been shown to be related to HLA antibody formation after kidney transplantation,^30^, ^31^ pregnancy,^32^ and pancreas and islet transplantation.^33^ We, therefore, analyzed the role of PIRCHE‐II and kidney graft failure in almost 3000 donor‐recipient couples. The natural logarithm of PIRCHE‐II was associated with a higher risk for graft failure, and the hazard ratio of graft failure for PIRCHE‐II numbers was significantly higher for first transplantations as compared with the overall cohort.^12^ The PIRCHE‐II algorithm might be used in the kidney donor selection procedure to provide additional information for risk stratification of specific donor‐patient couples and eventually lead to an improved graft survival.

## 3 NON‐HLA ANTIBODIES IN KIDNEY TRANSPLANTATION

In recent years, multiple studies have been published showing a relation between the presence of non‐HLA autoantibodies and graft loss and/or rejection episodes.^34^, ^35^, ^36^, ^37^ Although none of these studies have proven that autoantibodies actually contribute to the pathogenesis of graft dysfunction, the data indicate that detection of non‐HLA antibodies may be useful in identifying patients at risk for graft loss. This is acknowledged by companies producing reagents for HLA antibody detection which recently both developed a Luminex‐based multiplex assay for detection of non‐HLA antibodies. Reports are currently emerging on the clinical significance of these non‐HLA antibodies (or autoantibodies) using these commercial assays.^38^ In these reports, antibodies of the IgG isotype are measured, whereas it is possible that some non‐HLA antibodies relevant to prognosis are of another isotype and are missed. For example, in some autoimmune diseases, such as IgM‐RF in rheumatoid arthritis or IgA anti‐tTG in celiac disease, autoantibodies are of other isotypes. However, most of the clinically relevant autoantibodies in autoimmune diseases are of the IgG isotype.

Based on a literature search, we selected 14 proteins known to play a role in kidney function, and recognized by non‐HLA antibodies in renal disease.^39^ As extensive reviews on these antibodies have been published earlier,^34^, ^35^ their clinical relevance or first description are briefly mentioned below. Antibodies directed against targets expressed on the endothelium, that is, angiotensin II type 1 receptor (AT1R) and endothelin type A receptor (ETAR), were reported to be involved directly or indirectly in renal disease.^40^, ^41^, ^42^ The presence of antibodies against the glomerular basement membrane protein agrin has been associated with the number of rejections prior to diagnosis of transplant glomerulopathy,^43^ while pre‐transplant presence of antibodies reactive to peroxisomal‐trans‐2‐enoyl‐coA‐reductase (PECR) were associated with late development of transplant glomerulopathy.^44^ Pre‐ and post‐transplant antibody levels against the glomerular basement membrane protein endorepellin (C‐terminal part of perlecan) were increased in patients with vascular rejection.^45^ Antibodies against adipocyte plasma membrane‐associated protein (APMAP) and BPI fold‐containing family B member 1 (LPLUNC1) were found in sera of patients awaiting kidney retransplantation after nephrectomy of the first transplant.^46^ In a study where lymphocytic extracts of 20 healthy donors were incubated with sera of 28 chronic hemodialysis patients, antibodies against Rho GDP‐dissociation inhibitor 2 (ARHGDIB), Lamin B1, Tubulin beta‐4B (Tubb4B) and vimentin were showed.^47^ Autoantibodies against ARHGDIB were also described in acute leukemia patients.^48^ Production of anti‐vimentin antibodies after renal transplantation was significantly associated with interstitial fibrosis and tubular atrophy.^49^

Using a protein array with 5056 protein peptides, antibodies against Rho guanine nucleotide exchange factor 6 (ARHGEF6) were found using 36 paired pre‐ and post‐transplant sera of 18 pediatric renal transplant recipients.^50^ In another study using a similar approach, antibodies against protein kinase C zeta type (PRKCZ) were found in pediatric renal transplant recipients during allograft rejection.^51^ Phospholipase A2 receptor (PLA2R) antibodies are strongly associated with primary membranous nephropathy and are currently used as a diagnostic marker,^52^ although their role in the pathogenesis of recurrence of disease after kidney transplantation is not yet known.

In the PROCARE study, assays to detect non‐HLA antibodies against these 14 proteins were developed in‐house, where proteins were coupled either directly to beads or coupled via a HaloTag, to retain the 3D protein structure.^39^ All pre‐transplant sera from 4770 transplantations were analyzed with these reagents showing a heterogeneity in results with regard to median‐fluorescent intensities (MFI) for the various antigens.^53^ Commercial companies delivering reagents for autoantibody analyses (eg, for connective tissue diseases or transplantation) define cutoffs discriminating positive from negative sera on the basis of autoantibody levels in healthy donors. The rationale for this is that low concentrations of autoantibodies can be detected in each healthy individual. However, these cutoff definitions are a priori not always clinically relevant for kidney transplantation, as they are not defined for instance in relation with graft loss. To define the signal which is best related with graft loss, we analyzed MFI signals from all autoantibodies simultaneously for absolute MFI and signal‐to‐background ratios. This analysis was performed similar to a recent study yielding a sensible single antigen bead cut‐off for HLA antibodies with optimal association with graft loss.^8^ For each of these ratios, cutoffs, and their combinations, the difference in graft survival at year 1, 5, and 10 was calculated which maximally discriminates in graft loss between positive and negative patients. This analysis showed that especially autoantibodies against ARHGDIB appear to be clinically relevant in recipients transplanted with a kidney from a deceased donor (N = 3274) but not in recipients of a living‐donor kidney (N = 1496), corrected for recipient and donor age, type of donor, cold ischemia time for deceased donors (after brain or cardiac death), dialysis years, induction therapy with IL‐2 receptor blocker and presence of DSA. The results indicate that these autoantibodies are related with graft loss, while they occur independently from DSA.^53^ Therefore, pre‐transplant risk stratification could be improved by determining the anti‐ARHGDIB antibodies. Patients with these antibodies should be preferably transplanted with a living‐donor kidney, or, if this is not feasible, graft function should be monitored more closely signs of deterioration.

## 4 EFFECTOR MECHANISMS OF HLA AND NON‐HLA ANTIBODIES

### 4.1 Complement fixing donor‐specific HLA antibodies

Shortly after the introduction of single antigen bead assays to determine HLA antibodies, it became apparent that their results could not predict outcome of the classic complement‐ dependent cytotoxicity cross‐match. In addition, it was shown that not all IgG‐DSA detected by Luminex were indicative for a poor clinical prognosis. As the cross‐match detects complement‐fixing antibodies whereas the SAB technique detects all HLA‐binding IgG antibodies, this raised the question whether modification of the SAB technique into detection of complement‐fixing IgG could improve its ability to detect clinically relevant DSA. Modifications used to consist of using of IgG‐subclass specific conjugates instead of using a conjugate detecting pan‐IgG,^54^ or addition of human serum as a source of complement followed by detection of C3d,^55^ C1q^7^ or C4d^56^ fixation by appropriate conjugates.^57^ Activation of complement requires a minimal density of IgG bound to antigens.^58^ One of the key differences between HLA molecules present on beads vs cells is that HLA‐molecules are immovably fixed to beads whereas on the cell‐surface, HLA molecules float in the phospholipid bilayer and can be clustered rapidly by HLA‐antibodies leading to a clustered formation of IgG Fc‐tails. On beads, the detection of complement fixation is therefore a parameter of the amount of antibodies bound, irrespective of affinity or precise specificity, which explains the direct relation between MFI values found when detecting IgG vs deposition of complement components.^10^, ^59^, ^60^ In a small study with 34 kidney recipients, 19 with biopsy‐proven antibody‐mediated rejection (AMR) and 15 who did not have AMR, the C1q‐binding activity was shown to be largely due to differences in antibody strength (or higher MFI).^60^ They showed after dilution the C1q‐positive sera became negative and that 4‐fold concentration resulted in increased MFI levels in 4 of 6 C1q‐negative sera accompanied by a conversion to a C1q‐positive phenotype.^60^ Irrespective of the fact whether these in vitro assays reflect the capacity of an HLA antibody to fix complement in vivo, several recent studies show that the presence of complement activating DSA in vitro can be considered as a biomarker for risk stratification in solid organ transplantation.^61^, ^62^, ^63^, ^64^ A systematic review and meta‐analysis on 7936 solid organ transplants indeed confirmed that recipients with circulating complement‐activating DSA's experienced a more than 3‐fold increased risk of both allograft rejection and allograft loss compared with patients without complement fixing DSA's.^57^

### 4.2 Single nucleotide polymorphisms associated with IgG effector functions

The pathogenesis of endothelial damage from the transplanted kidney, caused by binding of HLA or non‐HLA antibodies to the cell surface, may involve complement activation, antibody dependent cellular cytotoxicity (ADCC), or intracellular signaling by clustering of target (eg, HLA) molecules.^65^ Single nucleotide polymorphisms (SNPs) in complement regulators and IgG effector functions were hypothesized to be important for effectiveness of complement or ADCC as different variants have been shown to be associated with kidney transplant outcome. C3 is the central complement component that by be activated by all three complement pathways.^66^ Expression of the allelic variants of the C3 allotype (rs2230199), slow (C3S) or fast (C3F), might affect the ability of C3 to interact with other complement receptors.^67^ In one study, C3S/S recipients of a C3F/F or C3F/S donor kidney had a better graft survival compared with recipients of C3S/S donor kidney,^68^ while in another larger study this could not be reproduced and no association was found between the C3 allotype and graft outcome.^69^ A number of polymorphisms in fluid phase regulators, such as Factor B (rs641153) and Factor H (rs800292, rs1061170, rs1065489), were related to the regulation of complement activity and therefore alter risk for age‐related macular degeneration.^70^ A link was found between Factor H SNP rs1065489 and effectiveness of rituximab treatment in patients with B‐cell lymphoma, as Factor H is a negative regulator of the complement pathway.^71^

HLA antibodies can induce sublytic quantities of C5b to C9 membrane attack complex on endothelial cells and kidneys tubular epithelial cells. CD59 inhibits formation of the membrane attack complex by preventing binding of C9 to C5b to C8 complexes. A SNP in the CD59 promotor region (rs147788946) of the donor was associated with higher risk for chronic rejection after lung transplantation.^72^ ADCC can be mediated by natural killer cells and to some extent monocytes and neutrophils. In this process, IgG bound to the cell surface is recognized via FcRγ receptors of which SNPs are known influencing the affinity for IgG, such as CD16a/FcγR3a (rs396991) and CD32/FcγR2a (rs1801274).^73^, ^74^, ^75^, ^76^

The configuration of these eight SNPs could potentially be used in pre‐transplant stratification to predict the degree of potential complement activity or ADCC activation and therefore might be related to the incidence or severity of rejection. CD59 is expressed by endothelial cells in the lung and kidney but also by other cells. We hypothesize that this CD59 SNP in the donor but not in the recipient is associated with kidney graft loss. Therefore, the CD59 SNP was determined both in the recipient and donor, as was C3. The other six SNPs were determined in the recipients only. These SNPs were studied in 205 transplantations having DSA as these gene polymorphisms are not expected to exert an effect in cases where DSA are absent (Figure 2). From the polymorphisms studied, especially the CD16a/FcγR3a SNP (rs396991) defining whether valine (V) or phenylalanine (F) is present at amino acid position 158 showed an inverse relationship with rejection‐free survival in the configuration with higher affinity for IgG (homozygous VV). This is consistent with previous publications showing improved responses to rituximab therapy in patients with lymphoma with VV compared with those with VF of FF at that position.^74^, ^77^

## 5 CONCLUSIONS

The conclusions from the PROCARE study are that nDSA antibodies detected by SAB assays are not associated with a lower graft survival. Both classes I and II SAB‐DSA are a clear risk factor for graft loss in deceased donor transplants, while in living donor transplants, only the combined presence of classes I and II SAB‐DSA may be associated with an increased risk for graft failure. The relevance of specific HLA‐epitopes recognized by B‐ and/or T‐cells is recognized in relation to kidney graft failure: (a) the number of pre‐transplant DESA might be a better parameter to stratify risk than the presence of serologically defined DSA and (b) PIRCHE‐II is associated with a higher risk for graft failure. Also, the presence of pre‐transplant antibodies against ARHGDIB was associated with increased risk for graft failure in deceased but not in living donor transplantations. These findings can be used in clinical practice for transplant risk stratification to improve graft and patient survival. At present, luminex assignment of DSA is being used in many HLA‐laboratories indicating that results can be included in an updated allocation algorithm. In a preliminary allocation, simulation on our cohort where a patient with DSA against HLA classes I and II would not be transplanted, the available kidney would be offered to the next patient on the wait list ranking. On the cohort level, this resulted in an increase of graft survival of this cohort and a lower chance of HLA‐immunization after transplantation. However, we also noticed that specific patient groups would suffer from a longer waiting time, indicating that any adjustment of the allocation system could not be beneficial for specific patient groups as has been appreciated in other studies on allocation.^78^, ^79^, ^80^, ^81^ For this reason, we will design adjustments of the allocation system that result in overall improvement of outcome, with minimization of inequalities in waiting time.

This would lead leading to a more efficient use of organ donors and a decrease in waiting time. However, adaptation of the current algorithm would not be directly beneficial for each patient on the waiting list, as for instance the waiting time for the highly immunized patients would not decrease.

## CONFLICT OF INTEREST

The authors have declared no conflicting interests.
